# Case report: First documented case of cerebral angiostrongyliasis caused by *Angiostrongylus costaricensis* in a free-ranging opossum

**DOI:** 10.3389/fvets.2024.1294484

**Published:** 2024-02-01

**Authors:** Tamara Solorzano-Scott, Fernando Aguilar-Vargas, Martha Cordero-Salas, Amanda Conejo, Alicia Rojas, Mario Baldi

**Affiliations:** ^1^Tropical Diseases Research Program, School of Veterinary Medicine, Universidad Nacional, Heredia, Costa Rica; ^2^Servicio de Patología Diagnóstica LAPAVET-ESFA, Escuela de Medicina y Cirugía Veterinaria San Francisco de Asís, Universidad Veritas, San José, Costa Rica; ^3^Servicio Nacional de Salud Animal, Ministerio de Agricultura y Ganadería, Heredia, Costa Rica; ^4^Widlife Veterinary Clinic, Santuario y Centro de Rescate Las Pumas, Cañas, Guanacaste, Costa Rica; ^5^Laboratory of Helminthology, Faculty of Microbiology, University of Costa Rica, San José, Costa Rica; ^6^Centro de Investigación en Enfermedades Tropicales, University of Costa Rica, San José, Costa Rica

**Keywords:** case report, parasitic diseases, angiostrongyliasis, wildlife reservoir, zoonosis

## Abstract

*Angiostrongylus costaricensis* is a metastrongyloid nematode that primarily infects the mesenteric arteries of wild rodents. This parasite is endemic in several regions of the American continent, and in humans, causes a disease known as abdominal angiostrongyliasis. Despite the important health implications of this nematode, there are limited studies investigating the involvement of wild animals in its life cycle. In this study, we present the clinical manifestations, pathologic findings, and molecular diagnosis, to the best of our current knowledge, of the first documented onset of cerebral angiostrongyliasis because of *A. costaricensis* infection in a juvenile free-ranging opossum (*Didelphis marsupialis*). Histopathological findings stress the presence of eosinophilic meningoencephalitis with nematodes present within the lesions, and PCR was positive for *cox*1 and ITS1 reactions. The obtained sequences for a 279 bp fragment of ITS1 were 100% identical to *A. costaricensis* from Costa Rica. This case highlights the substantial difficulties in diagnosing neuroangiostrongyliasis, yet underscores the importance of considering *A. costaricensis* as a potential culprit behind neurological conditions in wild marsupials. It acts as an urgent call to action to improve surveillance programs tracking infectious and parasitic diseases causing mortality in wildlife populations.

## 1 Introduction

*Angiostrongylus costaricensis* is a metastrongyloid nematode that primarily infects the mesenteric arteries of wild rodents. The life cycle of this parasite involves the infection of eight different taxonomic families of terrestrial gastropods as competent intermediate hosts, while definitive hosts include the cotton rat (*Sigmodon hispidus*) and other rodent species (1). During its development into the adult phase within vertebrates, *A. costaricensis* can take two distinct migratory routes. The primary route, referred to as the lymphatic-venous-arterial pathway, involves the migration of the worms through the lymphatic system and systemic arterial circulation until they establish their final niche within the mesenteric arteries. The hepatic-venous pathway, as a secondary route, solely documented in the context of experimental infections of *S. hispidus* (2).

Human beings typically serve as unintentional hosts due to the absence of egg deposition or L1 larval release into the intestinal lumen, a process that is characteristic of natural definitive hosts (3). Instead, the presence of parasite triggers a robust inflammatory response mediated by eosinophils, resulting in a disease known as abdominal angiostrongyliasis (4, 5). This condition is characterized by notable pathological features including marked infiltration of eosinophils into the intestinal wall, granulomatous formations, and eosinophilic inflammation of the blood vessels (vasculitis) most prominently affecting the ileocecal region of the intestine (1).

This parasite, first identified in Costa Rica in 1971, has attracted increasing attention due to reported cases in humans spanning a substantial geographic range throughout the American continent, with a notable concentration in Central and South America (1). Additionally, few cases reported in Africa and Europe imported from the Americas (6–9). In Costa Rica, a considerable number of children have been diagnosed with the disease, attributed to the consumption of mollusks hidden within vegetables (10).

A considerable proportion of wild species are prone to become infected through the ingestion of intermediate hosts (11). Nevertheless, despite the significant health implication of this nematode, there are limited studies investigating the involvement of wild animals in its life cycle, leaving this aspect of the community-epidemiology continuum unknown (1, 12, 13). Multiple cases of abdominal angiostrongyliasis due to *A. costaricensis* in wildlife species have been documented in the literature, including members of Procyonidae (raccoons), non-human primates and marsupials of the Didelphimorphia order (opossums) (14–16). Nevertheless, these reports have employed microscopic and histopathological techniques to identify parasites within lesions. Consequently, the verification of the diagnosis has proven unattainable. Subsequent research has revealed that certain cases were caused by Angiostrongylus species distinct from *A. costaricensis* (17).

Opossums belonging to the genus *Didelphis*, fulfill significant ecological functions, encompassing vital roles in seed dispersal as well as in the regulation of insect and gastropod populations (18). The common opossum, *Didelphis marsupialis* (Didelphidae), exhibits a broad geographic range across the Americas, spanning from Mexico to South America. Within Costa Rica, this species has been documented at elevations ranging from sea level to 2,000 meters above sea level, mainly occupying sites of high contact with human populations given their synanthropic nature (19).

Despite substantial evidence for *D. marsupialis* serving as a natural host for various zoonotic parasites, a survey of the published literature reveals no prior documented cases of neuroangiostrongyliasis attributable to *A. costaricensis* infection in this marsupial species (13). Hence, this study elucidates a noteworthy instance of neurological disease linked to infection caused by the parasite in a juvenile free ranging opossum (*Didelphis marsupialis*). This investigation entails a comprehensive analysis of clinical manifestations, pathological findings, and molecular diagnostics, revealing the potential epidemiological significance of the case.

## 2 Methods

This research, which focused on an animal that had already died naturally without any therapeutic intervention, in accordance with local legislation and institutional requirements, did not require ethical review or approval. This research was approved by the local wildlife authority, through permit R-SINAC-ACG-PI-026-2019.

**Figure 1:**
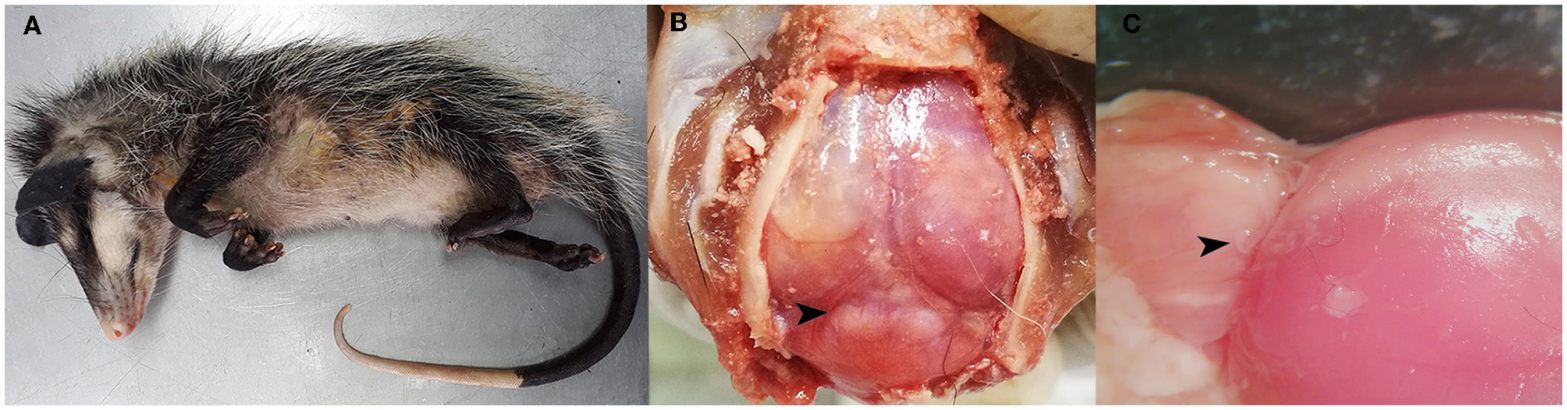
Post-mortem analysis findings. **(A)** Juvenile opossum (*Didelphis marsupialis*). **(B)** Multifocal areas of variable sizes, slightly raised and whitish in color, observed in the parietal lobes and occipital lobe of the brain, as well as in the cerebellar vermis (arrowhead). **(C)** Nematodes located in the leptomeninges above the cerebellar vermis and the occipital lobe (arrowhead).

A juvenile opossum (*Didelphis marsupialis*) exhibiting neurological symptomatology was discovered by agricultural laborers in a rural locality of the Guanacaste region, Costa Rica (10.501099° N, 84.9241900° W) (Figure 1A). Veterinary personnel transported the affected animal to a nearby wildlife rehabilitation facility for clinical evaluation. Upon examination, observed neurological signs included vestibular syndrome, disorientation, lethargy, and seizures. Regrettably, the opossum succumbed swiftly and naturally without any therapeutic intervention. Postmortem, the carcass was preserved via frozen storage at −20°C prior to being transported to the pathology laboratory for analysis.

This case was examined within the context of an initial pilot program designed to establish passive disease surveillance protocols within wildlife populations in Costa Rica. Macroscopic examination of all organs was performed following previously described protocols, with specimens preserved for histopathological assessment and relevant complementary tests conducted (20). These procedures considered both the clinical history and postmortem findings associated with the case. Brain samples were procured during the necropsy procedure and frozen at −20°C. Subsequently, sections of fresh brain tissue were meticulously affixed onto glass slides and subjected to microscopic scrutiny. The primary objective of this examination was to ascertain the potential presence of nematode parasites. Segments of the nematodes were subsequently isolated from the brain tissue and examined under a light microscope for further morphological and taxonomic analysis.

DNA was isolated from the retrieved portions of nematodes found within the brain parenchyma by using the DNeasy Blood & Tissue kit (Qiagen, Germany) according to the instructions of the manufacturer but eluting the purified DNA in 30 μl of elution buffer. Three different PCRs were run to amplify a fragment of the cytochrome oxidase subunit 1 (*cox*1) of the Phylum Nematoda and two independent reactions for amplifying the ITS1 loci of *Angiostrongylus* spp. and the phylum Nematoda. Accordingly, primers JB3 (5'-TTTTTTGGGCATCCTGAGGTTTAT-3') and JB4.5 (5'-TAAAGAAAGAACATAATGAAAATG-3') were used to amplify the 390 bp fragment of the *cox*1 with an initial denaturation step at 95°C, 35 cycles of amplification at 95°C for 1 min, 57°C for 1 min and 72°C for 45 s, and a final elongation step for 7 min (21).

A 400 bp fragment of the ITS-1 was amplified with rDNA2 (5'-TTGATTACGTCCCTGCCCTTT-3') and rDNA158S (5'-ACGAGCCGAGTGATCCACCG-3') (22) primers, following the previously described conditions (23). Finally, a PCR amplifying a 290 bp fragment of the ITS1 of *Angiostrongylus* spp. was run using primers AngioF1674 (5'-GTCGTAACAAGGTATCTGTAGGTG-3') and designed reverse primer AcosR1 (5'-GTCTATACGAGCGAACGCATAC-3') with an initial denaturation at 95°C, 35 cycles of 95°C for 1 min, 55°C for 1 min and 72°C for 1 min and a final denaturation step at 72°C (24). All amplicons were run in 1.5% agarose gels stained with SYBR-Safe. Positive reactions were purified using Exo-SAP and Sanger sequenced using the BigDye terminator cycle sequencing chemistry (Macrogen, South Korea). The acquired sequences underwent purification and were subsequently subjected to comparative analysis against the GenBank database.

In accordance with the established rabies protocols by the National Animal Health Service concerning animals displaying neurological symptoms, an analysis was conducted on brain tissue to detect the presence of rabies virus. Total RNA was extracted utilizing the commercially available DNeasy Blood and Tissue kit (Qiagen, Germany) following the manufacturer's standard protocol. The amplification of the nucleoprotein gene was conducted using the RT-PCR technique, employing the primers RAB504 (5′-TATACTCGAATCATGAATGGA GGTCGACT-3′) and RAB304 (5′-ACGCTTAACAACAACAARATCARAG-3′). The diagnostic procedure adhered to the established protocol (25, 26).

## 3 Results

Post-mortem analysis revealed poor overall body condition and ~5 mL of sanguineous abdominal effusion, further showed evidence of a mild catarrhal enteritis within the gastrointestinal tract. Nonetheless, the central nervous system exhibited the most remarkable lesions, characterized by multiple areas of diverse sizes that exhibited slight elevation and a whitish coloration. Lesions were evident upon examination of the parietal and occipital cerebral lobes, as well as within the cerebellar vermis (Figure 1B). After the removal of the dura mater, we observed a small and pale pink nematode associated with these lesions (Figure 1C). A subset of the postmortem analysis findings is depicted in Figure 1. Further examination of the remaining organ systems did not reveal any gross lesions.

**Figure 2:**
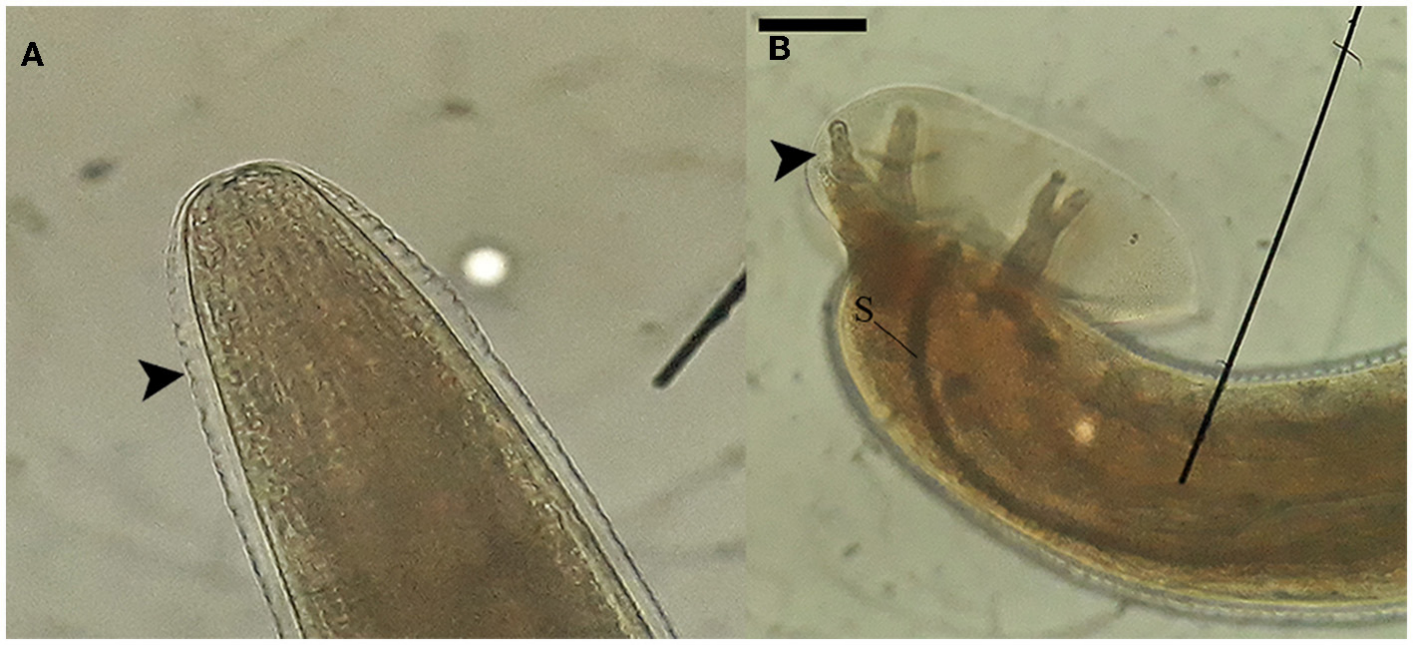
Morphological characteristics of nematodes collected from the neuroparenchyma. **(A)** The anterior end of the nematode possesses a rounded shape with a circular mouth opening. The cuticle of the nematode exhibits' transverse striations, as indicated by the arrowhead. **(B)** The posterior segment of a male nematode reveals the presence of the copulatory bursa, bursal rays (arrowhead), and the spicule (S). These images were captured using at a magnification of 100x, and the scale bar represents a length of 200 μm.

Nematode segments obtained from the brain tissue contained preserved sections of both the anterior and posterior regions of the worm. Specifically, the posterior segment exhibited distinctive features such as the copulatory bursa accompanied by bursal rays and the spicule, as visualized in Figure 2. These morphologic features enabled the distinction of these nematodes from other species that erratically migrate in mammalian brains (such as *Baylisascaris spp., Parelaphostrongylus spp., Elaphostrongylus spp*.) and placed them into the taxonomic order of Strongylida.

**Figure 3:**
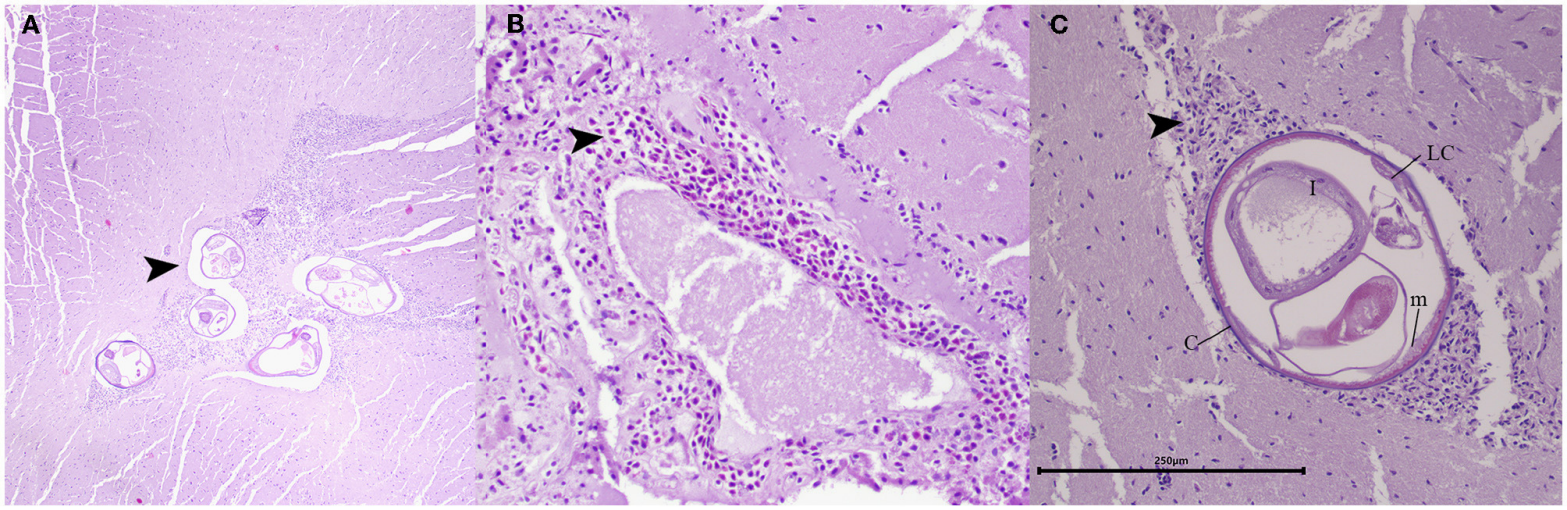
Histopathology of the cerebellum in juvenile opossum (*D. marsupialis*). **(A)** Lesions in the cerebellum: showing transverse sections of fully developed nematodes in the neuroparenchyma, affecting both gray matter and white matter, and surrounded by a mild mixed inflammatory infiltrate (arrowhead). H&E, magnification: 400x. **(B)** Lesions in pia mater: presenting a moderate inflammatory infiltrate, primarily composed of eosinophils, with a mild number of macrophages and lymphocytes (arrowhead). H&E, magnification: 200x. **(C)** The mature nematode: measuring approximately 250 μm in size, was characterized by a smooth cuticle (c), coelomyarian musculature (m), lateral cords (LC) and an intestine containing multinucleated cells (I). Encircling the nematode, a mixed inflammatory infiltrate comprising eosinophils, macrophages, and lymphocytes was discerned (arrowhead). H&E, magnification: 200x, scale bar: 250 μm.

During histopathological examination, cross sections of fully developed nematodes measuring ~250 μm (Figure 3A). These nematodes exhibited a smooth cuticle, polymyarian and coelomyarian musculature with lateral cords and an intestine containing multinucleated cells (Figure 3C). The nematodes were in the neuroparenchyma, affecting both gray matter and white matter of the cerebellum (Figure 3A), surrounded by a mild mixed inflammatory infiltrate consisting of eosinophils, macrophages, lymphocytes, and plasma cells. An analogous inflammatory infiltrate exhibited a perivascular pattern of distribution within the pia mater upon histological examination (Figure 3B). Despite the presence of various artifacts associated with specimen freezing, which hindered a comprehensive analysis of tissue damage, the findings suggested the presence of eosinophilic meningoencephalitis with nematodes present within the lesions.

PCR was positive for the *cox*1, and the ITS1 reactions. Despite this, distinct sequences were procured only for the 290-base pair ITS1 fragment. A 279 bp fragment of the ITS1 was obtained which showed 100% of identity to *A. costaricensis* (accession number GU58774) from Costa Rica with 99% of coverage. This sequence was 88.93% like *Angiostrongylus chabaudi* (accession number KM979214) and 89.44% similar to *Angiostrongylus vasorum* (accession number GU045374).

## 4 Discussion

Eosinophilic meningitis, caused by *Angiostrongylus cantonensis*, has well documented in wild animals (27). However, as far as our current knowledge is concerned, this represents the initial documented occurrence of cerebral angiostrongyliasis resulting from *A. costaricensis* infection. Previous studies have associated *A. costaricensis* infection in wild species with the development of granulomatous lesions in mesenteric arteries, as it normally occurs in humans (14, 15, 27). Prior case reports have documented an aberrant migration of this parasitic nematode within human testicular and hepatic tissues, eliciting accompanying inflammatory responses (8, 28). Despite the considerable diagnostic challenges posed by neuroangiostrongyliasis, previously reported cases in both humans and wild species, have consistently exhibited presumptive associations with *A. cantonensis* (27, 29).

ITS1 regions show high intra and interspecies variability in nematodes and other helminth species, making it a suitable marker for molecular identification (30). In the current study, A. *costaricensis* was identified with 100% identity and coverage and the next match was to A. *cantonensis* with 87.7% of identity, thus confirming our identification. Nevertheless, it should be highlighted that few *Angiostrongylus* spp. sequences are available in gene databases to allow comparisons between species, even though some research groups have made the efforts to deposit large datasets in Genbank (31). Therefore, molecular typing of collected *Angiostrongylus* spp. and other less-prevalent helminths is encouraged to increase the robustness of databases.

In experimental infections of *S. hispidus* with eggs of *A. costaricensis*, the normal migratory pathway in the rodent showed the presence of L3 and L4 larvae within blood vessels without associated inflammation in organs such as the kidney, lung, and brain after 9 days post-infection (2). Nevertheless, in this case, the identification of dead L4 larvae outside the blood vessels within the neuroparenchyma, accompanied by inflammation, suggests an aberrant migration, presenting a migratory pathway like to *A. cantonensis* (32, 33).

Even though an aberrant migration can occur due to proximate factors like a weakened host immune system rather than reflecting evolutionary adaptation. In this case, the inability to properly navigate the vascular system and susceptibility to immune attack in the CNS suggests the parasite lacks proximate adaptations to optimize infection in this host (34). Mechanisms to avoid immunogenic contact like surface molecular disguise, immunomodulation, or sensory and navigational changes have likely not yet evolved. While a weakened immune system could contribute, the overall evidence implies this parasite is still in early stages of adaptation to this host species (35). The aberrant migration and immune-mediated death of larvae point to inadequate proximate adaptations rather than a sole issue of host immunity (34).

Abdominal angiostrongyliasis has emerged as a noteworthy public health issue in Costa Rica, and its prevalence is often underestimated in other regions of the Americas. Nevertheless, the epidemiological significance of human infections is considered limited, as humans represent dead-end accidental hosts within the parasite's life cycle (7, 10). Despite the observation of inflammation in association with the presence of the parasite in the present case, without detectable eggs or L1 larvae in tissue or blood vessels, like humans, the migratory pathway of the parasite and the immune response of the opossum remain unclear. Thus, further extensive inquiries are justified to elucidate the role of opossums within the life cycle of *A. costaricensis* and establish its eco-epidemiological significance, especially considering its prevalence in both urban and rural localities and proximate associations with human and domestic animal population.

The mechanism of *A. costaricensis* exposure in this exceptional case of neuroangiostrongyliasis in a wild opossum remains unidentified. It is conceivable that the opossum might have consumed infected gastropods, given the frequent reports of human infections in the specific region where it discovered (10). Furthermore, the occurrence of the giant African snail (*Achatina fulica*) has been officially recorded within the vicinity of the area where the specimen was located (36). Although efforts make to control and eliminate this invasive species within the country, it well known that giant African snails are efficient intermediate hosts for various *Angiostrongylus* spp. and, consequently, should be consider a potential reservoir for *A. costaricensis*.

A differential diagnosis for neurological diseases observed in free-ranging marsupials should encompass the consideration of *A. costaricensis* as a potential etiological agent. Pharmacological and surgical management have proven effective in cases of aberrant migrations of *Angiostrongylus* spp. reported in domestic animals (37–39). The diverse migratory patterns of larvae within the host's body give rise to a wide spectrum of clinical signs, making resolution challenging in wild animals. This presents an extraordinary challenge in cases of cerebral angiostrongyliasis, where severe clinical symptoms manifest with limited therapeutic success despite extraordinary efforts (40, 41).

The diagnosis of neuroangiostrongyliasis presents challenges, particularly in wild animals, due to the lack of standardized diagnostic protocols. While serological tests are available for humans, diagnostic tests for veterinary species remain limited or untested (4). Consequently, it is probable that the identification of this disease will primarily rely on passive surveillance of severe cases and postmortem examinations, as exemplified in our case (42, 43). This leaves the establishment of preventive measures against exposure of animals in captivity and the control of intermediate hosts as the only viable options (44).

This case highlights the pressing need for a comprehensive One Health approach, emphasizing the initiative-taking measures required to bolster surveillance for infectious and parasitic causes of wildlife mortality. It underscores the critical connection between wildlife health and public health, emphasizing the importance of preventive strategies to mitigate potential implications on both fronts. Only through enhanced wildlife surveillance can we deepen our comprehension of the role played by these animals in the eco-epidemiology of pathogens. Such understanding is vital for assessing the risk of pathogen infections to humans and domestic animals, as well as the risks associated with the introduction of exotic parasites for the conservation of wildlife populations. Armed with this knowledge, we can implement preventive measures more effectively to mitigate these risks (45).

## Data availability statement

The original contributions presented in the study are included in the article/supplementary material, further inquiries can be directed to the corresponding author.

## Ethics statement

The requirement of ethical approval was waived by Ministeriode Ambiente y Energia, Costa Rica (MINAE), Sistema de Areas de Conservacion (SINAC) for the studies involving animals because we did work with wildlife carcass. The studies were conducted in accordance with the local legislation and institutional requirements. Written informed consent was obtained from the participants for the publication of this case report.

## Author contributions

TS-S: Conceptualization, Investigation, Methodology, Resources, Validation, Visualization, Writing—original draft, Writing—review & editing. FA-V: Conceptualization, Investigation, Methodology, Resources, Validation, Visualization, Writing—original draft, Writing—review & editing. MC-S: Investigation, Resources, Writing—review & editing. AC: Investigation, Writing—review & editing. AR: Investigation, Methodology, Resources, Validation, Writing—original draft, Writing—review & editing. MB: Conceptualization, Investigation, Resources, Validation, Writing—original draft, Writing—review & editing.
